# Vitamin D modifies the associations between circulating betatrophin and cardiometabolic risk factors among youths at risk for metabolic syndrome

**DOI:** 10.1186/s12933-016-0461-y

**Published:** 2016-10-06

**Authors:** Junling Fu, Cong Hou, Lujiao Li, Dan Feng, Ge Li, Mingyao Li, Changhong Li, Shan Gao, Ming Li

**Affiliations:** 1 Key Laboratory of Endocrinology, Department of Endocrinology, National Health and Family Planning Commission, Peking Union Medical College Hospital, Chinese Academy of Medical Sciences, Beijing, 100730 China; 2 Department of Endocrinology, Beijing Chaoyang Hospital, Capital Medical University, Beijing, 10043 China; 3 Department of Biostatistics and Epidemiology, University of Pennsylvania, Philadelphia, PA 19104 USA; 4 Division of Endocrinology, The Children’s Hospital of Philadelphia, Perelman School of Medicine, University of Pennsylvania, Philadelphia, PA 19104 USA

**Keywords:** Betatrophin, Vitamin D, Metabolic syndrome, Adolescents

## Abstract

**Background:**

Betatrophin has been recently reported to play a role in glucose homeostasis by inducing beta-cell proliferation in mice. However, studies in human are inconsistent. As a nutritionally-regulated liver-enriched factor, we hypothesize that betatrophin might be regulated by vitamin D, and ignorance of vitamin D status may explain the discrepancy in previous human studies. The aims of this study were to assess the association between circulating betatrophin and glucose homeostasis as well as other cardiometabolic variables in a cohort of youths at risk for metabolic syndrome and test the possible influence of vitamin D status on the association.

**Methods:**

559 subjects aged 14–28 years were recruited from Beijing children and adolescents metabolic syndrome study. All underwent a 2 h-oral glucose tolerance test. Serum levels of betatrophin, 25-hydroxy-vitamin D as well as adipokines including adiponectin and fibroblast growth factor 21 (FGF21) were measured by immunoassays. The relationships between betatrophin and insulin resistance, beta-cell function, other cardiometabolic variables and vitamin D status were evaluated.

**Results:**

Participants in the highest quartile of betatrophin levels had the highest levels of total cholesterol (*P* < 0.001), triglyceride (*P* < 0.001) and low-density lipoprotein cholesterol (*P* < 0.001) and the lowest levels of vitamin D (*P* = 0.003). After stratification by vitamin D status, betatrophin in subjects with vitamin D deficiency were positively correlated with unfavorable metabolic profiles including high blood pressures, dyslipidemia and hyperglycemia, whereas betatrophin in those with higher vitamin D levels only showed negative association with fasting insulin, 2 h-insulin, and insulin resistance. In addition, adiponectin and FGF21 demonstrated the expected associations with metabolic parameters.

**Conclusions:**

Elevated betatrophin levels were associated with cardiometabolic risk factors in this young population, but the association was largely dependent on vitamin D status. These findings may provide valuable insights in the regulation of betatrophin and help explain the observed discrepancies in literature.

**Electronic supplementary material:**

The online version of this article (doi:10.1186/s12933-016-0461-y) contains supplementary material, which is available to authorized users.

## Background

Betatrophin is a nutritionally-regulated secreted protein encoded by the *Gm6484* gene in mice and *C19orf80* in humans, and is expressed primarily in liver and adipose tissue [1]. Betatrophin was also named as RIFL (refeeding-induced fat and liver), Lipasin and angiopoietin-like protein 8 (ANGPTL8), and was initially implicated in lipid metabolism [2]. Recently Yi and colleagues found betatrophin can promote pancreatic beta-cell proliferation, improve glucose tolerance under insulin resistance (IR) and thus was named as betatrophin [3]. These results suggest that betatrophin not only plays a role in promoting glucose metabolism but also represents a promising drug target for replenishing beta-cell mass. Although later experimental studies challenged the initial indications about the role of betatrophin in regulation of beta-cell function [4–6], its role in regulating lipid metabolism has been confirmed by several other studies [7–9]. Despite the potential important metabolic role of betatrophin in mice, knowledge about betatrophin in humans is still inconsistent and controversial [10–24]. For instance, betatrophin levels were variable in type 2 diabetes mellitus (T2DM), either increased [11–14, 23] or decreased [15, 22], and similar patterns were observed in obesity [13, 15, 17–20] and IR [10–12, 15, 21, 22].

Despite the consensus on the involvement of betatrophin in lipid metabolism, the precise mechanism in this process remains unclear. The proposed mechanism is that betatrophin inhibits lipoprotein lipase (LPL) [25], and therefore in *Angptl8* knockout mice, the lower triglyceride (TG) phenotype is likely due to increased LPL activity [26]. Interestingly, vitamin D as one of the essential nutrients plays an important role in lipid metabolism, which is likely mediated by LPL pathway because it can induce LPL expression and increase LPL activity in adipocytes [27] and is positively associated with LPL concentration [28]. Moreover, previous studies indicated that betatrophin expression was regulated by nutritional intake [1], we thus hypothesize that vitamin D might also be served as a nutrient to regulate the expression and function of betatrophin. To date, no study has examined the role of vitamin D in mediating or modifying the association between betatrophin and metabolism. Thus, in the present study, we assessed the associations between circulating betatrophin levels and cardiometabolic risk factors including obesity, high blood pressure, dyslipidemia, IR, hyperglycemia and test whether these associations are affected by vitamin D conditions in a cohort of Chinese youth at risk for metabolic syndrome (MS).

Recent studies demonstrated that some adipokines, such as adiponectin, and fibroblast growth factor 21 (FGF21), were beneficial for improving metabolic effects [29]. However, few data are available regarding the relationship between betatrophin and these adipokines. Therefore, we also analyzed two well-known adipokines-adiponectin and FGF21 in similar models to assess their associations with cardiometabolic risk factors and to provide validity for our study.

## Methods

### Participants

A total of 559 subjects (14–28 years, mean = 20.2 years) were recruited from the cohort of Beijing children and adolescents metabolic syndrome (BCAMS) study. The BCAMS cohort study evaluated the prevalence of obesity and related metabolic abnormalities (hypertension, hyperglycemia, dyslipidemia) among a representative sample of Beijing school-age children (n = 19,593, ages 6–18 years, 50 % male) between April and October 2004 [30]. In this cohort, 4500 subjects were identified as having one or more of the following disorders: overweight defined by body mass index (BMI), increased total cholesterol (TC) ≥5.2 (mmol/L), TG ≥1.7 (mmol/L) or fasting glucose (FBG) ≥5.6 (mmol/L) based on finger capillary blood tests. We conducted follow up study after 10 years of initial investigation. Subjects were recruited consecutively over an 18-month period through various modalities and underwent medical examination in a center at Beijing Chaoyang Hospital. Signed informed consent was obtained from all participants and/or their parents or guardians through all the study processes. The BCAMS study was approved by the ethics committee at Beijing Chaoyang Hospital.

### Clinical and biochemical measurements

Subjects’ height, waist circumference (WC), weight, percent body fat, systolic and diastolic blood pressure (SBP and DBP) were measured by trained recruiters using standard methods. Participants removed bulky clothing and shoes prior to measurements. Height was measured to the nearest 0.1 cm using a portable stadiometer. WC was measured midway between the lowest rib and the top of the iliac crest. Weight and percent body fat were measured to the nearest 0.1 kg using a TANITA body composition analyzer (ModelTBF–300A). Measurements of right arm SBP and DBP were performed 3 times 10 min apart and the mean value of the latter two measurements was recorded. BMI was calculated as weight divided by height squared.

Venous blood samples were collected after an overnight (≥12 h) fasting. The samples were centrifuged, and immediately frozen for future analysis of hormones. A 2 h–75 g oral glucose tolerance test (OGTT) was performed on each subject. Serum lipids and glucose were assayed using the Hitachi 7060 C automatic biochemistry analysis system. Low density lipoprotein cholesterol (LDL-C) and high density lipoprotein cholesterol (HDL-C) levels were measured directly. HbA1c was assayed using the TOSOH G7 automatic analysis system. Serum aspartate transaminase (AST), alanine aminotransferase (ALT), creatinine and uric acid levels were assayed using automatic biochemistry analysis system and serum 25-hydroxy-vitamin D levels were measured by electro-chemiluminescence immunoassay with intra-assay and inter-assay coefficient of variations (CVs) of <7.5 and <6.8 %. Serum betatrophin levels were measured using enzyme-linked immunosorbent assay (ELISA) kits (WUHAN Eiaab Science; catalog number E11644 h) with intra- and inter- assay CVs of <8 and <10 %, respectively. The procedures were performed in accordance with the manufacturer’s instructions and the detection range was 78.0–5000 pg/ml, respectively. Insulin and adiponectin was measured by monoclonal antibody based ELISA [30] developed in the Key Laboratory of Endocrinology, Peking Union Medical College Hospital. Insulin assay had an inter-assay CV of <9.0 % and no cross-reactivity to proinsulin (<0.05 %). The intra-assay and inter-assay CVs for adiponectin were <5.4 and <8.5 %, respectively [31]. FGF21 was measured by Human Quantikine ELISA Kit (R&D Systems, Inc.) with intra- and inter-assay CVs of <4.8 and <7.4 %, respectively. All samples were analyzed in duplicates.

### Definitions and diagnostic criteria

IR was estimated using the following index: (1) homeostasis model assessment of IR (HOMA-IR) as fasting insulin(FINS) mU/L × FBG mmol/L/22.5; (2) insulin sensitive index (Matsuda Index) (ISI_M_), ISI_M_ = 10,000/(FBG × FINS) × (G × I), where G = mean serum glucose, and I = mean serum insulin concentration [32]. Pancreatic beta-cell function was assessed by (1) insulinogenic index (IGI = △Ins30/△Gluc30) and (2) oral disposition index (DIO = IGI × ISI) which was the product of insulin sensitivity and insulin secretion, yielded a better measure of beta-cell function [33]. The diagnosis of impaired fasting glucose (IFG), impaired glucose tolerance (IGT), prediabetes and T2DM profiles were based on the diagnostic criteria of American Diabetes Association [34]. IFG: FBG levels from 5.6 to 6.9 mmol/L; IGT: 2-h blood glucose (2 h-BG) levels from 7.8 to 11.0 mmol/L; prediabetes, IFG or IGT; T2DM: FBG ≥7.0 mmol/L or 2 h-BG ≥11.1 mmol/L. Nonalcoholic fatty liver disease (NAFLD) was diagnosed by B ultrasonography according to the 2010 Prevention and Treatment Guidelines for NAFLD published by the Society of Hepatology, Chinese Medical Association [35]. Vitamin D is deficient if ≤15 ng/mL [36].

MS in adolescents was defined by the presence of three or more of the following five components [30]: (1) central obesity: WC ≥90th percentile for age and gender in 10–16 years, WC ≥90 cm for boys and WC ≥80 cm for girls in over 16 years old; (2) elevated SBP/DBP ≥90th percentile for age, gender in 10–16 years, SBP ≥130 mm Hg or DBP ≥85 mm Hg for subjects over 16 years; (3) HDL-C <1.03 mmol/l in males, <1.29 mmol/l in females; (4) TG ≥1.70 mmol/l; (5) IFG defined as ≥5.6 mmol/L.

### Statistical analysis

Analyses were performed using Statistical Package for Social Sciences (SPSS) 19.0. A *P* value <0.05 (two-sided) was considered statistically significant. All skewed distributions were natural logarithmically transformed for analysis. Student’s t test and one-way ANOVA with Bonferroni’s post hoc comparisons were used for continuous variables. Partial correlation coefficients were calculated to evaluate the association between betatrophin and anthropometric measurements as well as other biomarkers associated with cardiovascular diseases.

## Results

Demographic and clinical characteristics of study subjects were listed in Table 1. The study includes 559 unrelated individuals recruited through the BCAMS study, 53 % of the participants were male, and the mean age was 20.2 ± 2.9 years. Compared to female subjects, male participants had higher BMI (*P* < 0.001), WC (*P* < 0.001), SBP (*P* < 0.001), DBP (*P* < 0.001), TG (*P* = 0.001), FBG (*P* = 0.001), 0.5 h-BG (*P* < 0.001) and HbA1c (*P* = 0.035) levels and lower HDL-C (*P* < 0.001) and DIO (*P* < 0.001) levels. As expected, females had higher adiponectin concentrations (*P* < 0.001) than males. However, there was no significant gender difference in FGF21. For betatrophin, the level in females was lower than that in males (*P* < 0.001).

**Table 1:** General characteristics of study subjects according to gender

Parameters	All	Male	Female	*P* Value
N (%)	559	294 (53 %)	265 (47 %)	/
Age (years)	20.2 ± 2.9	20.0 ± 3.0	20.4 ± 2.8	0.110
*Obesity traits*				
BMI (kg/m^2^)	25.7 ± 5.7	27.0 ± 5.8	24.3 ± 5.3	<*0.001*
WC (cm)	85.2 ± 14.6	90.5 ± 14.6	79.4 ± 12.1	<*0.001*
Percent body fat	30.4 ± 10.3	27.9 ± 9.4	33.2 ± 10.5	<*0.001*
*Pressures (mmHg)*				
SBP	114.7 ± 14.0	120.5 ± 13.8	108.2 ± 11.2	<*0.001*
DBP	73.2 ± 10.5	75.9 ± 10.3	70.2 ± 9.8	<*0.001*
*Lipids (mmol/l)*				
TC	4.35 ± 0.92	4.29 ± 0.86	4.41 ± 0.99	0.131
TG	1.13 ± 0.83	1.25 ± 1.01	1.01 ± 0.54	*0.001*
LDL-C	2.53 ± 0.79	2.56 ± 0.72	2.50 ± 0.86	0.371
HDL-C	1.44 ± 0.32	1.34 ± 0.28	1.54 ± 0.34	<*0.001*
*Glucose and insulin*-*related traits*				
Glucose_0_ (mmol/l)	4.92 ± 0.69	5.01 ± 0.86	4.82 ± 0.41	*0.001*
Glucose_30_ (mmol/l)	7.94 ± 1.51	8.17 ± 1.60	7.69 ± 1.36	<*0.001*
Glucose_120_ (mmol/l)	6.06 ± 1.84	6.17 ± 2.07	5.95 ± 1.54	0.179
HbA1c (%)	5.38 ± 0.48	5.42 ± 0.54	5.33 ± 0.40	*0.035*
Insulin_0_ (mU/L)^a^	1.94 ± 0.74	1.97 ± 0.75	1.89 ± 0.72	0.219
Insulin_30_ (mU/L)^a^	4.26 ± 0.72	4.27 ± 0.74	4.24 ± 0.71	0.636
Insulin_120_ (mU/L)^a^	3.61 ± 0.80	3.55 ± 0.86	3.67 ± 0.73	0.106
HOMA-IR^a^	0.41 ± 0.77	0.46 ± 0.77	0.35 ± 0.75	0.092
ISI_M_^a^	1.79 ± 0.65	1.76 ± 0.66	1.82 ± 0.64	0.278
IGI^a^	0.22 ± 0.81	0.16 ± 0.79	0.27 ± 0.82	0.127
DIO^a^	2.01 ± 0.77	1.93 ± 0.73	2.09 ± 0.81	*0.015*
*Hepar and renal*-*related traits*				
AST (IU/L)^a^	2.94 ± 0.32	3.02 ± 0.36	2.86 ± 0.25	<*0.001*
ALT (IU/L)^a^	2.96 ± 0.54	3.16 ± 0.55	2.75 ± 0.43	<*0.001*
Creatinine (umol/L)^a^	4.19 ± 0.23	4.32 ± 0.14	4.04 ± 0.21	<*0.001*
Uric acid (umol/L)^a^	5.84 ± 0.27	6.00 ± 0.21	5.66 ± 0.21	<*0.001*
*Adipokines*				
FGF21 (pg/ml)^a^	4.39 ± 1.12	4.39 ± 1.11	4.39 ± 1.13	0.986
Adiponectin (μg/mL)^a^	1.90 ± 0.65	1.75 ± 0.72	2.07 ± 0.53	<*0.001*
Betatrophin (pg/ml)^a^	5.77 ± 0.37	5.82 ± 0.36	5.71 ± 0.37	<*0.001*
Vitamin D (ng/ml)^a^	2.64 ± 0.41	2.76 ± 0.40	2.52 ± 0.38	<*0.001*

Values in italics are significant at *P* < 0.05

*BMI* body mass index; *WC* waist circumference; *SBP* Systolic blood pressure; *DBP* Diastolic blood pressure; *TC* total cholesterol; *TG* triglycerides; *LDL*-*C* low density lipoprotein cholesterol; *HDL*-*C* high-density lipoprotein cholesterol; *HOMA*-*IR* homeostasis model assessment for insulin resistance; *ISI*_*M*_ insulin sensitivity Matsuda index; *IGI* insulinogenesis index; *DIO* oral disposition index; *AST* aspartate aminotransferase; *ALT* alanine aminotransferase; *FGF21* fibroblast growth factor 21

^a^Skewed distributions were natural logarithmically transformed. Vitamin D was adjusting for visiting season. Data were expressed as *n* (%), mean ± SD. *P* values are from Student’s t test

To evaluate the relationships between betatrophin and cardiometabolic risk factors, participants were further divided into quartiles based on sex-standardized betatrophin levels. As shown in Table 2, subjects in the highest betatrophin quartile exhibited the highest levels of TC (*P* < 0.001), TG (*P* < 0.001) and LDL-C (*P* < 0.001) and the lowest levels of vitamin D (*P* = 0.003). However, no difference was observed for obesity traits, blood pressures, HDL-C, blood glucose/insulin traits, and adipokines with betatrophin levels.

**Table 2:** Relationship between sex-standardized betatrophin quartiles and cardiometabolic risk factors

Parameters	Betatrophin				*P* value
	Q1 (n = 138)	Q2 (n = 139)	Q3 (n = 138)	Q4 (n = 138)	
Male gender, n (%)	73 (52.9 %)	73 (52.5 %)	73 (52.9 %)	73 (52.9 %)	/
Age (years)	20.5 ± 3.1	20.0 ± 2.8	19.8 ± 2.9	20.4 ± 2.9	0.170
*Obesity traits*					
BMI (kg/m^2^)	25.5 ± 5.5	24.7 ± 5.2	25.9 ± 6.0	26.6 ± 5.9	0.051
WC (cm)	85.1 ± 13.8	83.1 ± 13.5	85.2 ± 15.5	87.3 ± 15.2	0.120
Percent body fat	30.1 ± 10.2	29.4 ± 9.5	30.2 ± 10.3	31.5 ± 10.4	0.346
*Pressures (mm* *Hg)*					
SBP	115 ± 14.9	113 ± 12.1	115 ± 13.5	116 ± 15.5	0.331
DBP	72 ± 10.7	73 ± 10.5	72.9 ± 9.5	75 ± 11.3	0.402
*Lipids (mmol/l)*					
TC	4.27 ± 0.82	4.19 ± 0.82	4.29 ± 0.79	4.65 ± 1.17*^,+,$^	<*0.001*
TG	0.98 ± 0.53	1.02 ± 0.44	1.17 ± 0.85*	1.37 ± 1.23*^,+^	<*0.001*
LDL-C	2.49 ± 0.71	2.40 ± 0.73	2.47 ± 0.66	2.77 ± 0.98*^,+,$^	<*0.001*
HDL-C	1.44 ± 0.29	1.42 ± 0.31	1.43 ± 0.35	1.45 ± 0.34	0.870
*Glucose and insulin*-*related traits*					
Glucose_0_ (mmol/l)	4.86 ± 0.51	4.97 ± 1.12	4.89 ± 0.44	4.95 ± 0.48	0.495
Glucose_30_ (mmol/l)	7.79 ± 1.33	7.99 ± 1.80	7.91 ± 1.42	8.05 ± 1.43	0.545
Glucose_120_ (mmol/l)	5.92 ± 1.30	6.01 ± 2.68	6.18 ± 1.45	6.15 ± 1.65	0.631
HbA1c (%)	5.34 ± 0.37	5.42 ± 0.66	5.35 ± 0.36	5.40 ± 0.48	0.515
Insulin_0_ (mU/L)^a^	1.91 ± 0.71	1.88 ± 0.66	1.96 ± 0.74	1.98 ± 0.84	0.665
Insulin_30_ (mU/L)^a^	4.30 ± 0.72	4.20 ± 0.63	4.31 ± 0.76	4.20 ± 0.78	0.460
Insulin_120_ (mU/L)^a^	3.61 ± 0.79	3.53 ± 0.79	3.61 ± 0.70	3.65 ± 0.90	0.661
HOMA-IR^a^	0.37 ± 0.75	0.36 ± 0.70	0.43 ± 0.75	0.46 ± 0.87	0.636
ISI_M_^a^	1.79 ± 0.65	1.83 ± 0.58	1.77 ± 0.65	1.79 ± 0.72	0.872
IGI^a^	0.29 ± 0.84	0.16 ± 0.76	0.26 ± 0.83	0.16 ± 0.80	0.423
DIO^a^	2.09 ± 0.87	2.00 ± 0.79	2.02 ± 0.65	1.93 ± 0.77	0.436
*Hepar and renal*-*related traits*					
AST (IU/L)^a^	2.91 ± 0.25	2.96 ± 0.35	2.94 ± 0.28	2.97 ± 0.39	0.412
ALT (IU/L)^a^	2.92 ± 0.49	2.94 ± 0.52	2.95 ± 0.53	3.04 ± 0.60	0.248
Creatinine (umol/L)^a^	4.17 ± 0.19	4.19 ± 0.23	4.19 ± 0.19	4.22 ± 0.28	0.333
Uric acid (umol/L)^a^	5.80 ± 0.26	5.83 ± 0.25	5.86 ± 0.30	5.88 ± 0.27	0.098
*Adipokines*					
FGF21 (pg/ml)^a^	4.41 ± 1.00	4.34 ± 1.03	4.44 ± 1.16	4.41 ± 1.30	0.914
Adiponectin (μg/mL)^a^	1.88 ± 0.65	1.94 ± 0.57	1.90 ± 0.68	1.89 ± 0.75	0.885
Betatrophin (pg/ml)^a^	5.37 ± 0.15	5.66 ± 0.09*	5.82 ± 0.08*^,+^	6.24 ± 0.43*^,+,$^	<*0.001*
Vitamin D (ng/ml)^a^	2.70 ± 0.36	2.70 ± 0.41	2.64 ± 0.35	2.53 ± 0.47*,^+^	*0.003*

Data were expressed as n (%), mean ± SD

One-way ANOVA where differences versus quintile 1 are indicated as * *P* < 0.05, differences versus quintile 2 are indicated as ^+^ *P* < 0.05, differences versus quintile 3 are indicated as ^$^ *P* < 0.05. Quartile values of betatrophin are expressed as Q1, Q2, Q3 and Q4 (pg/ml). For female: Q1, 127.5–41.8; Q2, 241.8–87.0; Q3, 287.0–56.9; Q4, 356.9–438.5. For male: Q1, 142.2–76.8; Q2, 276.8–29.3; Q3, 329.3–89.5; Q4, 389.5–475.6

Values in italics are significant at *P* < 0.05

*BMI* body mass index; *WC* waist circumference; *SBP* Systolic blood pressure; *DBP* Diastolic blood pressure; *TC* total cholesterol; *TG* triglycerides; *LDL*-*C* low density lipoprotein cholesterol; *HDL*-*C* high-density lipoprotein cholesterol; *HOMA*-*IR* homeostasis model assessment for insulin resistance; *ISIM* insulin sensitivity Matsuda index; *IGI* insulinogenesis index; *DIO* oral disposition index; *AST* aspartate aminotransferase; *ALT* alanine aminotransferase; *FGF21* fibroblast growth factor 21

^a^Skewed distributions were natural logarithmically transformed. Vitamin D was adjusting for visiting season

**Fig. 1:**
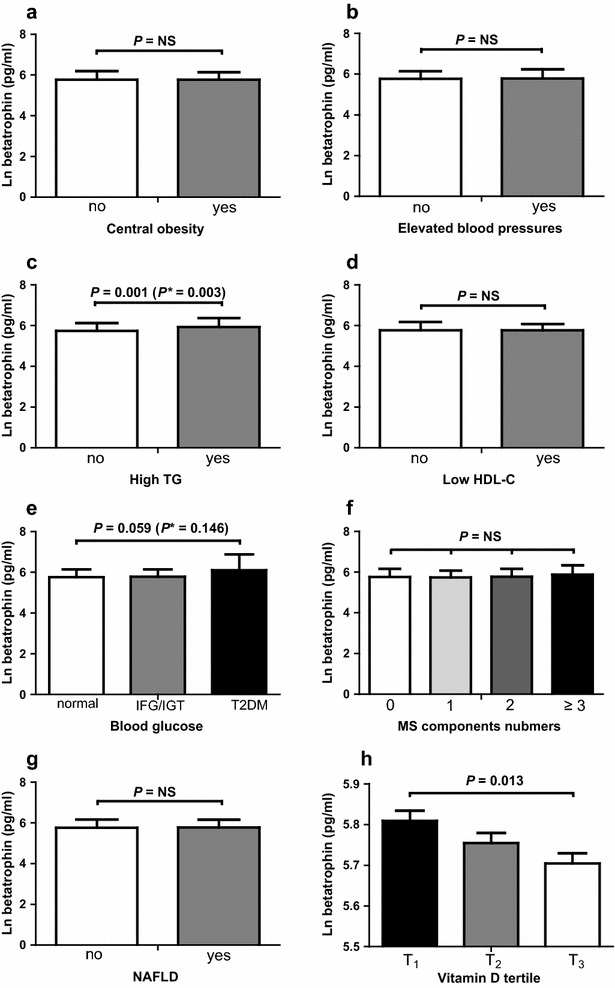
Levels of betatrophin in various metabolic abnormalities. Betatrophin concentrations were compared between subjects with and without **a** central obesity, **b** elevated blood pressures, **c** high TG, **d** low HDL-C and **g** NAFLD, and subjects with different **e** glucose tolerance status (normal, IGT/IFG, or T2DM, **f** number of MS components (0, 1, 2, or ≥ 3) and **h** vitamin D tertile ( T1, 3.00–11.94 ng/ml; T2, 11.94–17.04 ng/ml; and T3, 17.04–35.61 ng/ml). Data are natural log-transformed and shown as mean ± SEM. All *P* values were adjusted for gender and age and P* was further adjusted for vitamin D levels. *NS* no significant difference; *TG* triglycerides;* HDL*-*C* high-density lipoprotein cholesterol;* MS* metabolic syndrome;* IFG* impaired fasting glucose;* IGT* impaired glucose tolerance;* T2DM* type 2 diabetes mellitus;* NAFLD* nonalcoholic fatty liver disease

Next, we compared betatrophin levels across various metabolic abnormalities (Fig. 1). After controlling for age and gender, serum betatrophin concentrations were significantly increased in subjects with high TG levels (*P* = 0.001) and with lowest tertile of vitamin D levels (*P* = 0.013), and tended to be higher in T2DM (*P* = 0.059 vs. normal subjects; *P* = 0.137 vs. IFG/IGT). However, circulating betatrophin levels were not significantly different between those with and without central obesity, elevated blood pressures and NAFLD (all *P* > 0.05). In addition, betatrophin levels were not significantly different among the groups with different numbers of MS components (*P* > 0.05).

**Fig. 2:**
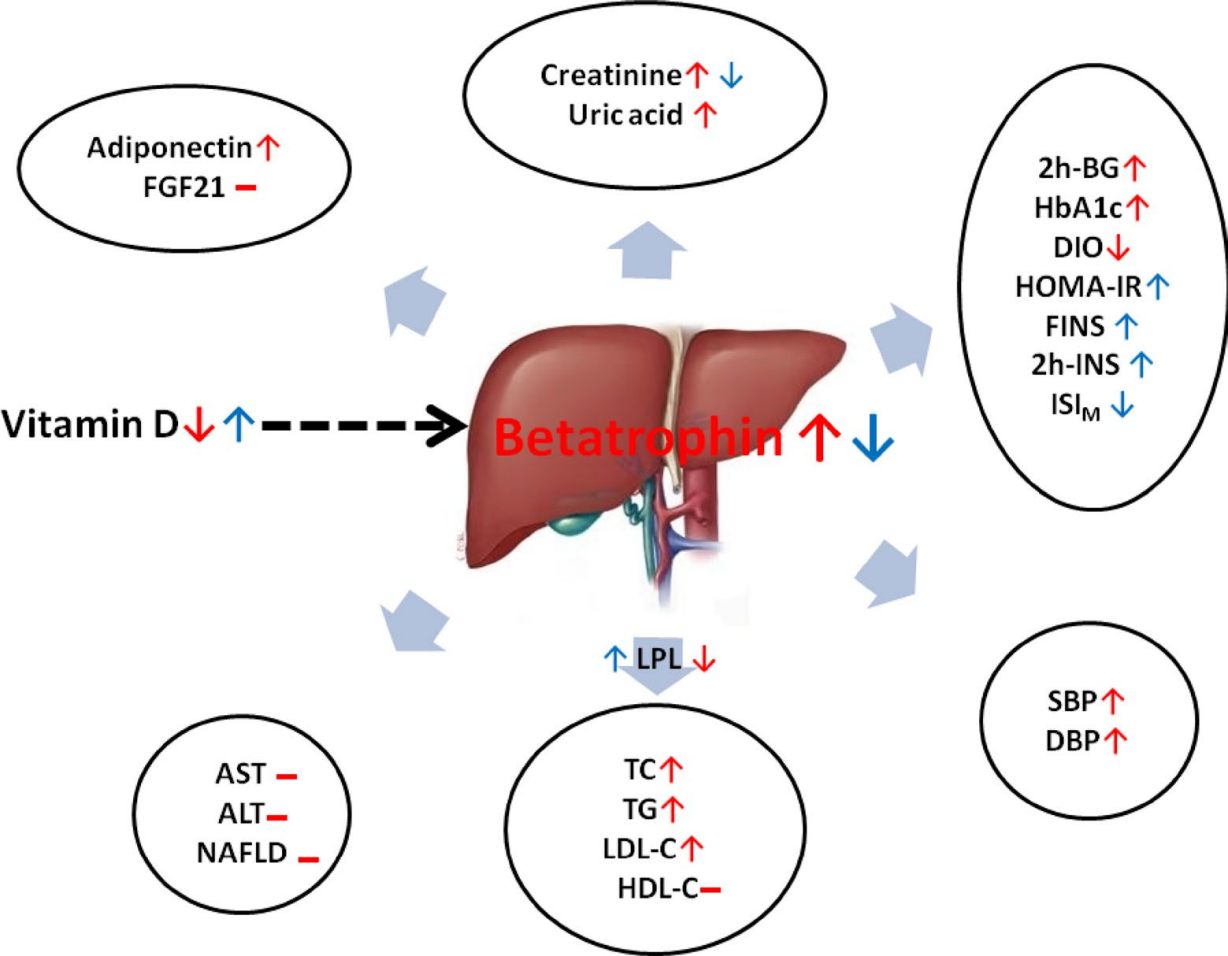
Possible associations between betatrophin and cardiometabolic variables and the influence of vitamin D status. *SBP* Systolic blood pressure; *DBP* Diastolic blood pressure; *LPL* lipoprotein lipase; *TC* total cholesterol; *TG* triglycerides; *LDL*-*C* low density lipoprotein cholesterol; *HDL*-*C* high-density lipoprotein cholesterol; *2h-BG* 2-hour blood glucose; *FINS* fasting insulin; *2h-INS* 2-hour insulin; *HOMA*-*IR* homeostasis model assessment for insulin resistance; *ISI*_*M*_ insulin sensitivity Matsuda index; *DIO* oral disposition index; *AST* Aspartate transaminase; *ALT* Alanine aminotransferase; *NAFLD* nonalcoholic fatty liver disease; *FGF21* fibroblast growth factor 21. *Color* in *red* indicates the associations with increased concentrations of betatrophin possibly induced by vitamin D deficiency; *color* in *dark blue* indicates the associations with low concentrations of betatrophin possibly inhibited by higher levels of vitamin D; *dotted black line* represents the possible role of vitamin D in regulating betatrophin levels; *N dash* means no significant association

In order to investigate whether the correlations between betatrophin and metabolic parameters were affected by vitamin D status, we further stratified participants into two groups according to vitamin D levels, as shown in Table 3. In the whole-population, betatrophin levels were negatively correlated with vitamin D (*P* < 0.001), and positively correlated with TC (*P* < 0.001), TG (*P* = 0.024), LDL-C (*P* = 0.002), HbA1c (*P* = 0.005), as well as creatinine (*P* < 0.001), uric acid (*P* = 0.001) and adiponectin (*P* = 0.044). No association was observed between betatrophin and measures of adiposity. In the vitamin D deficient group (vitamin D ≤ 15 ng/ml), we observed more significant correlations with above mentioned variables. Additionally, betatrophin levels were positively correlated with SBP (*P* = 0.004), DBP (*P* = 0.001), 2 h-BG (*P* = 0.016) and negatively correlated with DIO (*P* = 0.025). However, these associations were not observed in the vitamin D > 15 ng/ml group except for creatinine (*P* = 0.024). Nonetheless, in the vitamin D > 15 ng/ml group, betatrophin was positively correlated with ISI_M_ (*P* = 0.019) and negatively related with FINS (*P* = 0.023), 2 h-INS (*P* = 0.019), and HOMA-IR (*P* = 0.038). Figure 2 summarizes the associations between betatrophin and cardiometabolic variables and the possible influence of vitamin D levels.

**Table 3:** Age- and gender- adjusted partial correlations coefficients between Ln-betrathrophin and metabolic parameters stratified by vitamin D status

Variables	All (n = 559)		Vitamin D > 15 ng/ml (n = 250)		Vitamin D ≤ 15 ng/ml (n = 309)	
	*r*	*P*	*r*	*P*	*r*	*P*
*Obesity traits*						
BMI (kg/m^2^)	0.033	0.435	0.024	0.708	0.044	0.442
WC (cm)	0.014	0.736	−0.034	0.593	0.064	0.270
Percent body fat	−0.004	0.917	−0.012	0.856	0.002	0.978
*Pressures (mmHg)*						
SBP	0.026	0.537	−0.119	0.063	0.163**	*0.004*
DBP	0.075	0.079	−0.041	0.519	0.186**	*0.001*
*Lipids (mmol/l)*						
TC	0.155***	<*0.001*	0.048	0.456	0.271***	<*0.001*
TG^a^	0.096*	*0.024*	−0.001	0.993	0.170**	*0.003*
LDL-C	0.134**	*0.002*	0.018	0.780	0.247***	<*0.001*
HDL-C	0.022	0.611	0.076	0.236	−0.013	0.818
*Glucose and insulin*-*related traits*						
Glucose_0_ (mmol/l)	0.041	0.331	0.048	0.456	0.036	0.531
Glucose_30_ (mmol/l)	0.070	0.109	0.018	0.786	0.107	0.067
Glucose_120_ (mmol/l)	0.074	0.090	−0.044	0.504	0.140*	*0.016*
HbA1c (%)	0.119**	*0.005*	0.074	0.246	0.161**	*0.005*
Insulin_0_ (mU/L)^a^	−0.025	0.551	−0.145*	*0.023*	0.079	0.173
Insulin_30_ (mU/L)^a^	−0.043	0.321	−0.097	0.138	0.003	0.953
Insulin_120_ (mU/L)^a^	−0.058	0.183	−0.153*	*0.019*	0.040	0.498
HOMA-IR^a^	−0.016	0.709	−0.133*	*0.038*	0.084	0.144
ISI_M_^a^	0.040	0.360	0.154*	*0.019*	−0.063	0.287
IGI^a^	−0.083	0.060	−0.092	0.165	−0.077	0.192
DIO^a^	−0.059	0.183	0.019	0.773	−0.133*	*0.025*
*Hepatic and renal*-*related traits*						
AST (IU/L)^a^	0.040	0.345	0.068	0.289	0.020	0.723
ALT (IU/L)^a^	0.043	0.317	0.009	0.894	0.087	0.131
Creatinine (mg/dL)^a^	0.228***	<*0.001*	0.144*	*0.024*	0.319***	<*0.001*
Uric acid (mg/dL)^a^	0.136**	*0.001*	0.118	0.065	0.165**	*0.004*
*Adipokines*						
FGF21 (pg/ml)^a^	0.004	0.933	−0.063	0.336	0.058	0.324
Adiponectin (μg/mL)^a^	0.086*	*0.044*	0.039	0.545	0.142*	*0.013*
Vitamin D (ng/ml)^a^	−0.196***	<*0.001*	−0.069	0.306	−0.265***	<*0.001*

Values in italics are significant at *P* < 0.05

*BMI* body mass index; *WC* waist circumference; *SBP* Systolic blood pressure; *DBP* Diastolic blood pressure; *TC* total cholesterol; *TG* triglycerides; *LDL*-*C* low density lipoprotein cholesterol; *HDL*-*C* high-density lipoprotein cholesterol; *HOMA*-*IR* homeostasis model assessment for insulin resistance; *ISI*_*M*_ insulin sensitivity Matsuda index; *IGI* insulinogenesis index; *DIO* oral disposition index; *AST* Aspartate transaminase; *ALT* Alanine aminotransferase; *FGF21* fibroblast growth factor 21

^a^Skewed distributions were natural logarithmically transformed. Vitamin D was adjusting for visiting season. r: Partial Correlation Coefficients

* *P* ≤ 0.05

** *P* ≤ 0.01

*** *P* ≤ 0.001

Further, we examined the relationships between adiponectin and FGF21 using similar models (Additional file 1: Table S1). As expected, FGF21 levels were positively correlated with obesity traits (*P* < 0.001), blood pressures (*P* < 0.001), lipid profiles and unfavorable blood glucose/insulin traits (all *P* < 0.05), whereas adiponectin had the opposite correlations with metabolic profiles compared with FGF21, but these associations were not modified by the way of vitamin D influencing on betatrophin.

## Discussion

In this young population with risk for MS, we demonstrated that increased betatrophin levels were associated with higher lipids (i.e. TG, TC and LDL-C) even after controlling for BMI, but no significant difference was observed in subjects with and without central obesity, high blood pressure, MS and NAFLD. Our novel finding is that circulating betatrophin was increased in subjects with vitamin D deficiency and the associations with blood pressures, lipids, glucose/insulin traits, creatinine and uric acid were largely dependent on vitamin D status. In addition, this study replicated the well-known negative association of adiponectin and positive association of FGF21 with MS-related risk factors [29].

### The associations of betatrophin and cardiometabolic variables without consideration of vitamin D status

Betatrophin was initially implicated in lipid metabolism, as expected, our study confirmed the previous findings that circulating betatrophin was positively correlated with TG, TC and LDL-C, but not with HDL-C [7–9]. Besides its role in lipid regulation, betatrophin is also expected to play as a novel hormone to regulate glucose homeostasis in human as its function in mice [3]. However, results from adult clinical studies were controversial, for example, some studies reported that circulating betatrophin levels were positively correlated with HbA1c [11, 14] and increased in T2DM [11–14], while others exhibited decreased betatrophin concentrations in T2DM as well as obesity [15, 22]. Furthermore, two recent studies focus on children and adolescents also reported controversial results, betatrophin levels either decreased [20] or increased in obesity children with IR [10]. In our study, we found betatrophin was positively correlated with HbA1c, but only slightly elevated in T2DM patients compared to normoglycemic and prediabetic subjects, although the numbers of the young T2DM (9 cases) were too small to make a safe conclusion. However, no significant difference existed between individuals with and without obesity or MS. These discrepancies might be due to different study populations, or the use of different ELISA kits that recognize different terminus of betatrophin [37]. However, here we showed that another explanation is the confounding due to vitamin D status.

### The associations of betatrophin and cardiometabolic variables by vitamin D status

The effects of vitamin D on glucose and lipid metabolism have been investigated extensively in recent years [38]. Low vitamin D levels, have been found to contribute to various CVD risk factors, such as hypertension, atherosclerosis, coronary artery disease and stroke [39]. In this study, we found vitamin D levels were negatively associated with circulating betatrophin concentration, and subjects in the lowest tertile of vitamin D levels had the highest betatrophin levels. Previous studies have indicated that vitamin D plays an important role in the lipid metabolism by inducing LPL expression and increasing LPL activity in adipocytes [27], and the positive correlation between vitamin D and LPL levels were also confirmed in a population study [28]. Interestingly, betatrophin is also referred to as Lipasin for its LPL inhibition effect. Given these observations, we speculated that vitamin D might be a major regulator of betatrophin expression, and they may also interact with each other, involved not only in lipid metabolism through the pathway of LPL, but also in other cardiometabolic regulation. Therefore, we further analyzed the associations between betatrophin and lipid/glucose parameters, and other cardiometabolic risk factors through stratifying the participants into two groups according to vitamin D levels. As we expected, the associations between betatrophin and cardiometabolic parameters depend largely on vitamin D status (as summarized in Fig. 2).

Firstly, we found the correlations between betatrophin and lipid profiles are more significant in the vitamin D deficiency group and disappeared in the higher levels of vitamin D group. The possible mechanisms between betatrophin and lipid profiles are as follows: Firstly, both betatrophin and vitamin D play important roles in lipid metabolism. Betatrophin inhibits LPL activity to elevate TG levels, whereas vitamin D stimulates LPL activity to decrease TG levels, they coordinate with each other to keep a balance of lipid metabolism. Subjects with vitamin D deficiency display a reduction of LPL activity and a compensatory secretion of betatrophin and then lead to the increasing of serum lipids levels and this effect disappeared when vitamin D is not deficient. Secondly, while it is not possible to determine causality with the present design, available data have shown that vitamin D is hydroxylated in the 25 position to yield 25-hydroxyvitamin D in liver, meanwhile, betatrophin mainly expressed in liver; we thus speculate that there might exist a direct reciprocal down-regulation between those two hormones in liver; however, further experimental study with focus on their direct interaction in liver may help explain the findings in current study. In addition, it should be noted that a few other studies found no correlation between betatrophin and lipid profiles [10, 11, 21]. In view of these inconsistent results, the underlying mechanism needs to be further studied in future studies beyond the possible pathway mentioned above.

For glycometabolism, in the vitamin D deficiency group, betatrophin was positively correlated with HbA1c and 2 h-BG levels, and negatively correlated with DIO levels, whereas in subjects with higher levels of vitamin D, betatrophin levels were negatively associated with insulin and HOMA-IR levels, and positively correlated with ISI_M_ levels. As mentioned above, the expression of betatrophin may be up-regulated under the vitamin D deficiency status whereas suppressed under the high levels of vitamin D levels. Thus, at least in the present study, we did not observe the promising role of betatrophin in promoting insulin secretion or improving glucose tolerance as reported in mice [3]; conversely, we found increased betatrophin levels were associated increased HbA1c and glucose under vitamin D deficiency status; while a negative association between betatrophin levels and HOMA-IR or insulin levels were only observed under higher vitamin D levels, which in line with a recent study including 75 children [20]. Since vitamin D deficiency was very common in patients with IR or T2DM, thus, we speculated that vitamin D deficiency might responsible for the elevated betatrophin in T2DM or IR in previous reports [11–14].

Additionally, we found serum betatrophin concentrations were positively correlated with blood pressures in vitamin D deficiency group. The positive associations between betatrophin and blood pressures were reported in patients with GDM [40] or T2DM [41]. In addition, previous meta-analysis demonstrated a significant inverse association between circulating 25(OH)D levels and risk of incident GDM [42] and T2DM [43]; and lower 25-hydroxyvitamin D levels were associated with higher blood pressure levels and increased rates of incident hypertension [44]. Thus, the positive correlation between betatrophin and blood pressures in GDM or T2DM might also be attributed to vitamin D deficiency. However, since no other study is available, further studies are warranted to validate our findings.

In the present study, creatinine and uric acid are strongly positively correlated with betatrophin levels and the correlations are more significant in vitamin deficient group. In contrast, a cross-sectional study of 148 pregnant patients [45] found no correlation between circulating betatrophin levels and the markers of renal function. The potential mechanisms supporting the associations should be elucidated by further studies. Meanwhile, betatrophin is mainly expressed in liver and strongly associated with TG, therefore, circulating betatrophin levels are supposed to correlate with NAFLD [18]. However, we found no association between betatrophin and NAFLD, as well as the aminotransferase levels which have also been demonstrated to be a marker of the severity of NAFLD.

### The associations of betatrophin and adipokines

Our data suggest that circulating betatrophin levels are not correlated with FGF21 levels, but positively associated with adiponectin concentrations. Like betatrophin, FGF21 produced preferentially from liver, and function in multiple tissues, with beneficial metabolic effects such as reduction of body weight and liver fat, improvement of insulin sensitivity, antihyperglycemic, antihyperlipidemic and thermogenic properties [46]. The two hepatokines (betatrophin and FGF21) are not correlated with each other, suggesting their potential production and function mechanisms may be different. Adiponectin is the most abundant peptide secreted by adipocytes and is considered a potent modulator of lipid and glucose metabolism with antidiabetic, antiatherogenic and anti-inflammatory properties, and plays an important role in the pathogenesis of metabolic diseases [46]. Since there are no published studies on the relationship between betatrophin and adiponectin, further studies should be conducted to investigate the mechanism of our findings.

Our study is the first that accounts for vitamin D condition when assessing the relationships between betatrophin and cardiometabolic risk factors. Additionally, the replication of expected associations between important adipokines (adiponectin and FGF21) and metabolic measurements provides validity and power in accordance with the methodology used here.

We recognize that this study has several limitations. The cross-sectional nature of this study does not allow the examination of temporal association or causality. Although the different biological relationships observed between betatrophin and variables of metabolism in different vitamin D nutritional status are likely universal, our sample was limited to a particular group of Chinese youths, thus, our findings may not be simply generalized to other populations. Further large sample studies in different ethnicities will be necessary to verify the relationships between betatrophin, vitamin D condition and cardiometabolic risk factors and find the direct evidences to elucidate the regulatory pathway. In addition, the wide age range of our study population and the lack of puberty information in those of 14–18 years old adolescents should also be recognized as the limitation, thus, to minimize this limitation, we adjusted for age, and sex when performing analysis.

In summary, our study demonstrated that betatrophin levels were increased with vitamin D deficiency. Furthermore, the associations between betatrophin and cardiometabolic risk factors including high blood pressures, dyslipidemia, and hyperglycemia were largely influenced by vitamin D status and were found exclusively among subjects with vitamin D deficiency. We believe that the lack of explanation for inconsistent results in the literature is due to the ignorance of vitamin D status, and further mechanistic studies are required to confirm this finding.

## Additional file

10.1186/s12933-016-0461-y Age- and gender-adjusted partial correlation coefficients between FGF21, adiponectin and metabolic parameters stratified by vitamin D status.

## Abbreviations

MS: metabolic syndrome
IFG: impaired fasting glucose
IGT: impaired glucose tolerance
NAFLD: nonalcoholic fatty liver disease
BMI: body mass index
WC: waist circumference
SBP: systolic blood pressure
DBP: diastolic blood pressure
LPL: lipoprotein lipase
TC: total cholesterol
TG: triglycerides
LDL-C: low density lipoprotein cholesterol
HDL-C: high-density lipoprotein cholesterol
HOMA-IR: homeostasis model assessment for insulin resistance
ISI_M_: insulin sensitivity Matsuda index
IGI: insulinogenesis index
DIO: oral disposition index
AST: aspartate transaminase
ALT: alanine aminotransferase
FGF21: fibroblast growth factor 21
