# Supplementary material for: Vitamin D modifies the associations between circulating betatrophin and cardiometabolic risk factors among youths at risk for metabolic syndrome
Source: Cardiovasc Diabetol. 2016 Oct 6;15:142. doi: 10.1186/s12933-016-0461-y (PMC5054537; doi:10.1186/s12933-016-0461-y)
Supplement: Supplementary file 1 — 10.1186/s12933-016-0461-y Age- and gender-adjusted partial correlation coefficients between FGF21, adiponectin and metabolic parameters stratified by vitamin D status. [file 12933_2016_461_MOESM1_ESM.docx]

| **Supplementary Table 1.** Age- and gender- adjusted partial correlation coefficients between FGF21, adiponectin and metabolic parameters stratified by vitamin D status | | | | | | | | | | | | |
| --- | --- | --- | --- | --- | --- | --- | --- | --- | --- | --- | --- | --- |
| **Variables** | **Ln-FGF21** | | | | | | **Ln-APN** | | | | | |
|  | **All** | | **Vitamin D > 15ng/ml**  **(n = 250)** | | **Vitamin D ≤ 15ng/ml**  **(n = 309)** | | **All** | | **Vitamin D > 15ng/ml**  **(n = 250)** | | **Vitamin D ≤ 15ng/ml**  **(n = 309)** | |
|  | ***r*** | ***P*** | ***r*** | ***P*** | ***r*** | ***P*** | ***r*** | ***P*** | ***r*** | ***P*** | ***r*** | ***P*** |
| **Obesity traits** | | | | | | | | | | | | |
| BMI (kg/m^2^) | 0.293*** | **< 0.001** | 0.300*** | **< 0.001** | 0.283*** | **< 0.001** | -0.391*** | **< 0.001** | -0.346*** | **< 0.001** | -0.432*** | **< 0.001** |
| WC (cm) | 0.298*** | **< 0.001** | 0.312*** | **< 0.001** | 0.283*** | **< 0.001** | -0.369*** | **< 0.001** | -0.323*** | **< 0.001** | -0.414*** | **< 0.001** |
| Percent body fat | 0.255*** | **< 0.001** | 0.279*** | **< 0.001** | 0.236*** | **< 0.001** | -0.353*** | **< 0.001** | -0.295*** | **< 0.001** | -0.403*** | **< 0.001** |
| **Pressures (mmHg)** | | | | | | | | | | | | |
| SBP | 0.275*** | **< 0.001** | 0.329*** | **< 0.001** | 0.229*** | **< 0.001** | -0.230*** | **< 0.001** | -0.210** | **0.001** | -0.251*** | **< 0.001** |
| DBP | 0.277*** | **< 0.001** | 0.280*** | **< 0.001** | 0.270*** | **< 0.001** | -0.208*** | **< 0.001** | -0.183** | **0.004** | -0.222*** | **< 0.001** |
| **Lipids (mmol/l)** | | | | | | | | | | | | |
| TC | 0.210*** | **< 0.001** | 0.235*** | **< 0.001** | 0.205*** | **< 0.001** | 0.019 | 0.649 | -0.087 | 0.176 | 0.093 | 0.105 |
| TG ^#^ | 0.392*** | **< 0.001** | 0.377*** | **< 0.001** | 0.393*** | **< 0.001** | -0.315*** | **< 0.001** | -0.272*** | **< 0.001** | -0.329*** | **< 0.001** |
| LDL-C | 0.308*** | **< 0.001** | 0.304*** | **< 0.001** | 0.323*** | **< 0.001** | -0.147** | **0.001** | -0.242*** | **< 0.001** | -0.085 | 0.140 |
| HDL-C | -0.249*** | **< 0.001** | -0.188** | **0.004** | -0.281*** | **< 0.001** | 0.414*** | **< 0.001** | 0.362*** | **< 0.001** | 0.438*** | **< 0.001** |
| **Glucose and insulin–related traits** | | | | | | | | | | | | |
| Glucose_0_ (mmol/l) | 0.087* | **0.044** | 0.091 | 0.164 | 0.085 | 0.145 | -0.103* | **0.016** | -0.165* | **0.010** | -0.076 | 0.185 |
| Glucose_30_ (mmol/l) | 0.144** | **0.001** | 0.197** | **0.003** | 0.109 | 0.065 | -0.110* | **0.011** | -0.220** | **0.001** | -0.041 | 0.480 |
| Glucose_120_ (mmol/l) | 0.211*** | **< 0.001** | 0.239*** | **< 0.001** | 0.201** | **0.001** | -0.111* | **0.010** | -0.297*** | **< 0.001** | -0.027 | 0.642 |
| HbA1c (%) | 0.158*** | **< 0.001** | 0.128* | **0.050** | 0.176** | **0.003** | -0.071 | 0.095 | -0.183** | **0.004** | -0.015 | 0.789 |
| Insulin_0_ (mU/L) ^#^ | 0.236*** | **< 0.001** | 0.171** | **0.009** | 0.285*** | **< 0.001** | -0.368*** | **< 0.001** | -0.343*** | **< 0.001** | -0.400*** | **< 0.001** |
| Insulin_30_ ( (mU/L) ^#^ | 0.156*** | **< 0.001** | 0.191** | **0.004** | 0.126* | **0.034** | -0.217*** | **< 0.001** | -0.203** | **0.002** | -0.226*** | **< 0.001** |
| Insulin_120_ ( (mU/L) ^#^ | 0.246*** | **< 0.001** | 0.276*** | **< 0.001** | 0.216*** | **< 0.001** | -0.258*** | **< 0.001** | -0.347*** | **< 0.001** | -0.181** | **0.002** |
| HOMA-IR ^#^ | 0.240*** | **< 0.001** | 0.177** | **0.007** | 0.286*** | **< 0.001** | -0.366*** | **< 0.001** | -0.349*** | **< 0.001** | -0.391*** | **< 0.001** |
| ISI_M_ ^#^ | -0.263*** | **< 0.001** | -0.222** | **0.001** | -0.291*** | **< 0.001** | 0.360*** | **< 0.001** | 0.357*** | **< 0.001** | 0.368*** | **< 0.001** |
| IGI ^#^ | 0.042 | 0.348 | 0.069 | 0.306 | 0.016 | 0.789 | -0.138** | **0.002** | -0.067 | 0.314 | -0.187** | **0.001** |
| DIO ^#^ | -0.176*** | **< 0.001** | -0.121 | 0.074 | -0.222*** | **< 0.001** | 0.147** | **0.001** | 0.221** | **0.001** | 0.104 | 0.079 |
| **Hepar and renal-related traits** | | | | | | | | | | | | |
| Creatinine (mg/dL) ^#^ | 0.127** | **0.003** | 0.096 | 0.142 | 0.151** | **0.009** | 0.095* | **0.025** | -0.013 | 0.837 | 0.176** | **0.002** |
| Uric acid (mg/dL) ^#^ | 0.247*** | **< 0.001** | 0.254*** | **< 0.001** | 0.236*** | **< 0.001** | -0.293*** | **< 0.001** | -0.329*** | **< 0.001** | -0.255*** | **< 0.001** |
| AST (IU/L) ^#^ | 0.107* | **0.013** | 0.147* | **0.024** | 0.080 | 0.170 | -0.090* | **0.034** | -0.206** | **0.001** | 0.018 | 0.758 |
| ALT (IU/L) ^#^ | 0.196*** | **< 0.001** | 0.226*** | **< 0.001** | 0.175** | **0.003** | -0.210*** | **< 0.001** | -0.252*** | **< 0.001** | -0.183** | **0.001** |
| **Adipokines** | | | | | | | | | | | | |
| FGF21 (pg/ml) ^#^ | / | / | / | / | / | / | -0.210*** | **< 0.001** | -0.181** | **0.005** | -0.218*** | **< 0.001** |
| Adiponectin (μg/mL) ^#^ | -0.210*** | **< 0.001** | -0.181** | **0.005** | -0.218*** | **< 0.001** | / | / | / | / | / | / |
| Betatrophin ^#^ | 0.004 | 0.933 | -0.063 | 0.336 | 0.058 | 0.324 | 0.086* | **0.044** | 0.039 | 0.545 | 0.142* | **0.013** |
| **Vitamin D ^#^** | -0.026 | 0.561 | 0.073 | 0.288 | -0.080 | 0.170 | -0.039 | 0.370 | 0.068 | 0.314 | -0.051 | 0.381 |

^#^ Skewed distributions were natural logarithmically transformed. Vitamin D was adjusted for visiting season. *r*: Partial Correlation Coefficients. ^*^ *P* ≤ 0.05, ^**^*P* ≤ 0.01, ^***^ *P* ≤ 0.001.

Abbreviations: BMI, body mass index; WC, waist circumference; SBP, Systolic blood pressure; DBP, Diastolic blood pressure; TC, total cholesterol; TG, triglycerides; LDL-C, low density lipoprotein cholesterol; HDL-C, high-density lipoprotein cholesterol; HOMA-IR, homeostasis model assessment for insulin resistance; ISI_M_, insulin sensitivity Matsuda index; IGI, insulinogenesis index; DIO, oral disposition index; AST, Aspartate transaminase; ALT, Alanine aminotransferase; FGF21, fibroblast growth factor 21.

Note: Values in bold are significant at *P* ≤ 0.05.
